# Experimental investigation on pore characteristics of vitrain and durain in low rank coal based on fractal theory

**DOI:** 10.1038/s41598-024-55668-4

**Published:** 2024-02-29

**Authors:** Chao Zheng, Yue Chen, Lan Yu, Wulin Lei, Xuanhong Du, Fengfeng Yang

**Affiliations:** 1 College of New Energy, Long Dong University, Qingyang, 745000 China; 2 College of Geology and Environment, Xi’an University of Science and Technology, Xi’an, 710054 China

**Keywords:** Coal, Natural gas

## Abstract

The macro petrographic compositions and its pore characteristics of coal reservoir play critical role in the accumulation and development of coalbed methane (CBM). In this paper, the pore characteristics of vitrain and durain were analyzed through the experiment and fractal theory. The results indicated that the micropores and microfractures develop in vitrain, and that transitional pores develop in durain. The pore volume and specific surface area (SSA) of vitrain are larger than those of durain, with the micropore SSA of vitrain being 35% higher than that of durain. The threshold pressure and tortuosity of vitrain are greater than that of durain, but the mean pore size of vitrain is smaller than that of durain. The fractal dimension *D*_1_ of vitrain is greater than that of durain, while the fractal dimension *D*_2_ is opposite, indicating that the pore surface of vitrain is coarser, and the pore structure of durain is more complex. The fractal dimension *D*_k_ of vitrain is larger than that of durain, the mean fractal dimension *D*_s_ of vitrain is smaller than that of durain, which shows that the diffusivity of vitrain is weak but the seepage capacity is strong due to the developed fractures. The difference in material composition and pore characteristics between vitrain and durain provides a new understanding for the development of CBM in low rank coal.

## Introduction

Coalbed methane (CBM) is a by-product of coal formation that is widely used due to its clean and low-carbon energy properties^1–3^. CBM is stored in the pore and fracture system of the coal reservoir in multiple occurrence states^4–6^. Hence, the pore development characteristics of coal are the crucial factors for the adsorption/desorption capacity of CBM^7–10^. To date, a large number of studies have been conducted on the pore characteristics of coal using multi-scale testing and analysis methods^11–14^, and numerous research results have been achieved^15–18^. Through literature review, it was found that the main influencing factors controlling the pore development and structural characteristics of coal reservoir are the degree of metamorphism, chemical structure, maceral components, tectonic action etc^17,19–21^. As the coal metamorphism degree increases, the macropores decrease and the micropores develop^22^. Due to the low proportion of aromatic nuclei in low-rank coal, the high proportion of functional groups and long side chains, the spatial structure of coal reservoir is loose. With increasing the aromatic nuclei, the side chains gradually decompose and the length decreases, so that the structure becomes more compact^23^. Vitrinite showed a strong positive correlation with micropores and pore specific surface area, while inertinite showed a negative correlation^24^. In different coal ranks, macerals have different effects on adsorption capacity^25^. The pore structure of tectonic coal is more complex than that of primary coal, suggesting that tectonic action changed the adsorption/desorption capacity of methane through its influence on the pore distribution^26^. In order to intuitively describe the occurrence states and migration characteristics of methane in different pore sizes, Hu et al.^27^ proposed new pore classification method, i.e. inaccessible pores (< 0.38 nm), filling pores (0.38–1.5 nm), diffusion pores (1.5–100 nm), and seepage pores (> 100 nm). Nano-scale micropores provide adsorption sites for methane, while nano-scale mesopores and all micro-scale pores have no adsorption energy for CH_4_^28^, the most important factors affecting methane adsorption capacity were the volume and specific surface area of ultra-micropores^8^.

The pore structure of coal is very complex and its pore distribution and surface topography are heterogeneous. It is difficult to quantitatively describe their complex pore characteristics using traditional euclidean geometric theory^29^. However, the pore structure of coal shows self-similarity within a certain scale, and its spatial distribution pattern is between two and three dimensions. Like most natural rocks, they have fractal characteristics^30^ and are more suitable for fractal geometric description^31^. As a tool to describe complex phenomena, fractal theory provides a new scientific method for studying the complex pore structure of coal^32^. The fractal characteristics of the coal pore structure correlate with the heterogeneity and complexity of the pore structure with its adsorption and seepage capacity of methane, micropores are the primary causes of heterogeneity^33^. That is, by analyzing the relationship fractal dimension and coal rank, coal composition and pore structure, and revealed the influence of pore structure on CH_4_ adsorption and seepage capacity^34^. Fractal dimension *D*_2_ displayed a positive correlation with SSA, and the negative correlation with mean pore size^35^. Coal reservoir with more complex pore surfaces and simpler pore structures has stronger methane adsorption capacity^36^, and the coalification makes coal surfaces and pore networks comparatively smoother and more regular for lower rank coals, but rougher and more complex for higher rank coals^37^. The width that corresponds to the inflection points of the fractal curves are considered to be the critical value for determining the pores and fractures in coal^38^.Therefore, it is very important to quantitatively analyze the fractal characteristics of pore structure by using fractal dimension.

These studies form the basis for further investgation on the pore structure of coal and adsorption/desorption of CBM. It must be noted that the macro petrographic composition has different effects on adsorption/desorption and migration of methane^39–44^, but litter research has been done on the pore characteristics of macro petrographic composition. In this study, the pore characteristics of vitrain and durain (two typical macro petrographic composition) were investigated by mercury intrusion porosimetry (MIP), low-pressure nitrogen adsorption (LP-N_2_) and low-pressure carbon dioxide (LP-CO_2_). Based on the test data, the appropriate evaluation model of pore fractal characteristics were selected to analyze the difference of pore fractal characteristics between vitrain and durain, which fully reflects the surface roughness of micropores, pore complexity of mesoporous pores, and seepage and diffusion capacity of macropores in the same coal sample. It provides a new perspective for studying methane adsorption, storage and desorption-transport capacity of different macro petrographic composition in low-rank coal reservoirs, which is helpful to actively promote the efficient development of CBM in low-rank coal.

## Experiments and methods

### Sample preparation

In this study, coal samples come from 12102 working face of Yuanzigou mine (YZG) in Yonglong mine area and 20418 working face of Huangling No. 2 mine (HL) in Huangling mine area of Huanglong coal field. YZG-JM and HL-JM represent vitrain, and YZG-AM and HL-AM represent durain. According to GB/T 482-2008, large block coal samples were collected from the working face, and the size of 1.0 cm sample was used for MIP. The stripped vitrain and durain fragments were ground into powder with size of 0.18–0.25 mm, the proximate analysis, LP-N_2_ adsorption and LP-CO_2_ adsorption were conducted. The particle size < 1.0 mm were selected for maceral identification.

### Experimental test method

AutoPore IV 9500 was used for mercury intrusion porosimetry (MIP). The pore size range is 0.005–1000 μm, and the reporting range is 0.10–61,000.00 psia. The volume accuracy of MIP is better than 0.1uL. The ASAP 2020 (V4.03) equipment was selected for low pressure nitrogen adsorption/low pressure carbon dioxide adsorption (LP-N_2_/ LP-CO_2_). Analysis parameters: specific surface area 0.0005 m^2^/g, pore size analysis 3.5–5000 Å, resolution 0.2 Å, minimum pore volume detection 0.0001 ml/g, and N_2_ temperature is 77 K. The principle of LP-CO_2_ adsorption is similar to that of LP-N_2_ adsorption. Compared with N_2_, CO_2_ gas molecules are smaller, diffusion rate is faster, and there is a higher saturation pressure at 273.15 K, which can be used for fine characterization of micropores.

### Evaluation method of pore fractal characteristics

The pore structure of coal plays a crucial role in the adsorption/desorption of CBM, and the fractal model can well characterize the roughness and the complexity of pore structure^45^. At present, the main methods to calculate the fractal dimension of pores based on LP-N_2_ adsorption data include fractal BET model, fractal FHH model and thermodynamic model. Among them, FHH (Frenkel-Halsey-Hill) proposed by PFEIFER et al^46^ is widely used in the calculation of fractal dimension of pore structure characteristics of porous materials due to its convenient calculation. Its calculation formula (1) is as follows:

1 $$\ln V = (D - 3)\ln \left[ {\ln (p_{0} /P)} \right] + C$$

where $P$ is adsorption capacity of gas at equilibrium pressure *P*, cm^3^/g; $p_0$ is saturated vapor pressure of gas, MPa; $C$ is constant; $D$ is fractal dimension. Therefore, with $\ln \left[ {\ln (p_{0} /P)} \right]$ as the horizontal coordinate, and $\ln V$ as the vertical coordinate, the curve slope can be obtained through linear fitting, and the fractal dimension can be scored.

MIP can well characterize macropores in coal reservoirs, so the Washburn equation is the basis for calculating the fractal dimension by MIP. There are mainly Menger sponge model, Capillary model, Sierpinski model and Thermodynamic model for calculating pore fractal dimension of MIP^47^. Jia Tengfei et al.^45^ evaluated the pore fractal model of low-rank coal reservoirs and found that there are certain differences in the characterization of fractal dimension obtained by different models. The relationship between fractal dimension and pore structure parameters shows that Menger sponge synthesis model and capillary synthesis model have limited parameters and insufficient precision. The Sierpinski model can well characterize the pore morphology of coal reservoir. Based on the relationship between pressure and mercury intrusion amount, the model is obtained^45^, the calculation formula as (2):

2 $$\ln (V) = \ln \alpha + (3 - D)\ln (P - P_{c} )$$

where $V$ is mercury intrusion amount (mL/g); $\alpha$ is constant; $P$ is mercury intrusion pressure (MPa); $P_c$ is the threshold pressure (MPa).

## Results and discussion

### Material composition characteristic

The low rank coal is in the primary stage of coalification, the results of coal proximate analys were shown that there are some differences in material composition between vitrain and durain (Table 1). The volatile yield of coal sample is high (22.28–35.57%), in which volatile yield of vitrain is higher than that of durain. However, the fixed carbon and ash yield of durain are greater than that of vitrain, and the difference of fixed carbon is relatively small. The difference in material composition is the result of the evolution of coal-forming environment.

**Table 1:** Results of proximate analysis.

Coal sample	Sample type	Moisture (%)	Ash (%)	Volatile (%)	Fixed carbon (%)
YZG-JM	Vitrain	4.21	1.38	35.57	58.86
YZG-AM	Durain	5.31	6.75	25.97	62.05
HL-JM	Vitrain	2.84	3.21	34.65	59.38
HL-AM	Durain	4.46	9.80	22.28	63.71

### Maceral components characteristic

The maceral components test results of coal samples showed that vitrain is rich in vitrinite, and the content of vitrinite ranges from 64.5 to 78.6%, with an average of 71.05%. Durain is rich in inertinite, and the content of inertinite ranges from 54.1 to 74.6%, with an average of 67.37%. The content of liptinite in vitrain and durain generally low, about 1.2% on average (Table 2). The difference of maceral components between vitrain and durain is an important reason for determining pore development.

**Table 2:** The results of maceral components.

Coal sample	Sample type	Vitrinite (%)	Inertinite (%)	Liptinite (%)	Mineral (%)
YZG-JM	Vitrain	86.51	8.45	2.24	2.80
YZG-AM	Durain	30.50	64.15	0.95	4.40
HL-JM	Vitrain	90.26	6.96	0.78	2.00
HL-AM	Durain	24.73	66.14	0.73	8.40

### Micropore development characteristics of vitrain and durain

**Figure 1:**
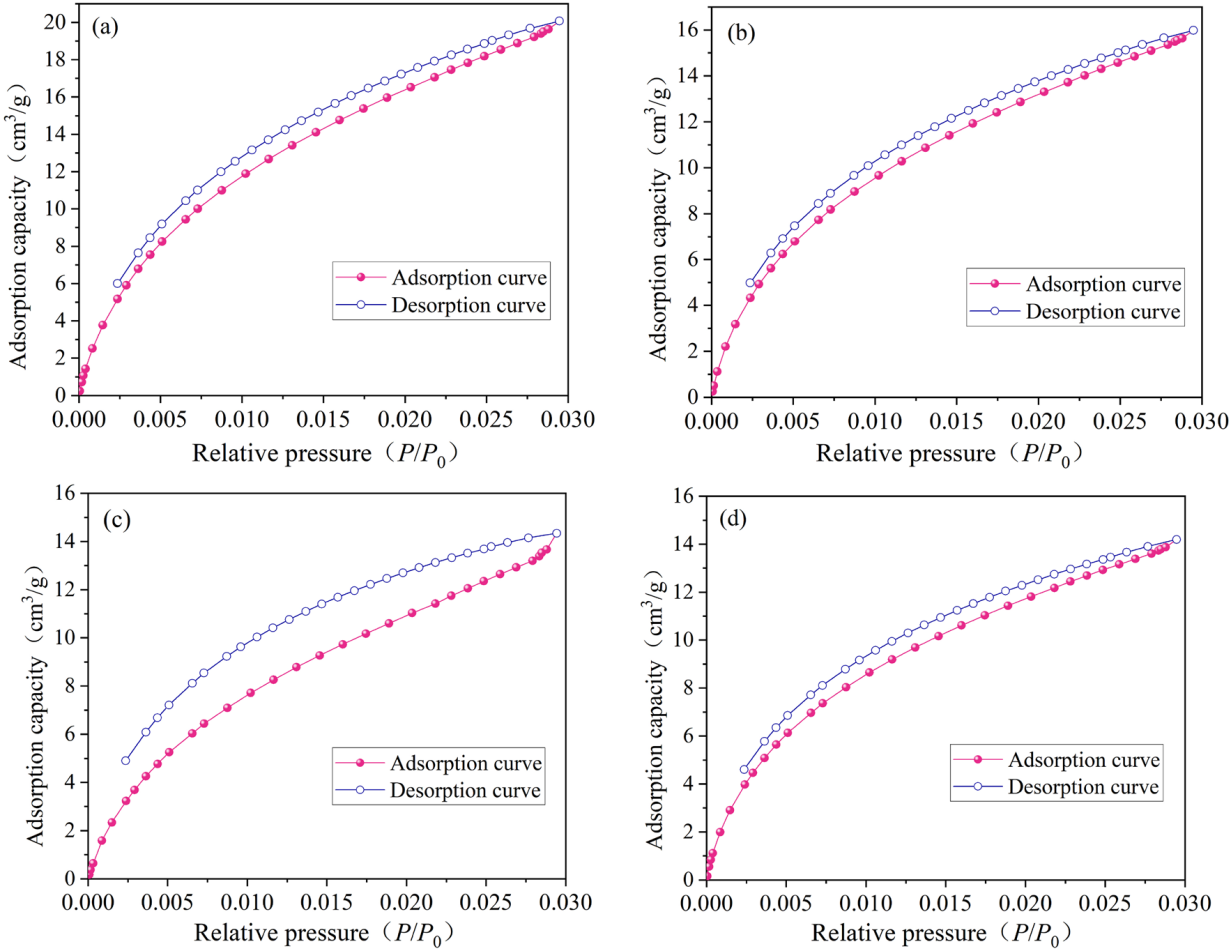
LP-CO_2_ adsorption/desorption curves of vitrain and durain (**a**) YZG-JM, (**b**) YZG-AM, (**c**) HL-JM, (**d**) HL-AM.

According to the pore classification standard of IUPAC^48^, micropores is pore size < 2 nm. Because of LP-N_2_ adsorption can only measure the pore size > 1.8 nm, so the micropore characterization result are not accurate. Compared with N_2_ molecules, CO_2_ molecules are smaller and can enter smaller pores, which can be reflected more objectively micropores (< 2 nm). The LP-CO_2_ absorption/desorption were analyzed by DFT model recommended by ISO and IUPAC^48^. The LP-CO_2_ absorption/desorption curves of vitrain and durain were shown in Fig. 1.

As shown in Table 3, BET pore specific surface area (SSA) of YZG-JM and YZG-AM are 150.737 m^2^/g and 114.937 m^2^/g, respectively, and those of HL-JM and HL-AM are 139.559 m^2^/g and 101.589 m^2^/g, respectively. The DFT pore SSA of YZG-JM and YZG-AM are 160.51 m^2^/g and 127.417 m^2^/g, respectively, while those of HL-JM and HL-AM are 117.623 m^2^/g and 113.924 m^2^/g, respectively. As a whole, the calculation results of the two models showed that the SSA of vitrain is larger than that of durain, and the calculation results of the two models have little difference, ranging from 6.25 to 15.8%. The BET mean pore size of YZG-JM and YZG-AM are 0.975 nm and 1.018 nm, respectively, and that of HL-JM and HL-AM are 0.752 nm and 1.023 nm, respectively. In other words, the mean pore size of durain is larger than that of vitrain.

**Table 3:** Pore characteristics of vitrain and durain with LP-CO_2_ adsorption.

Coal sample	BET micropore SSA (m^2^/g)	BET mean pore size (nm)	DFT micropore SSA (m^2^/g)		
			> 0.384 (nm)	> 1.066 (nm)	0.384 ~ 1.066 (nm)
YZG-JM	150.737	0.975	160.51	75.339	85.171
YZG-AM	114.937	1.018	127.417	56.338	71.079
HL-JM	139.559	0.752	117.623	65.943	51.680
HL-AM	101.589	1.023	113.924	49.502	64.422

### Mesopore development characteristics of vitrain and durain

**Figure 2:**
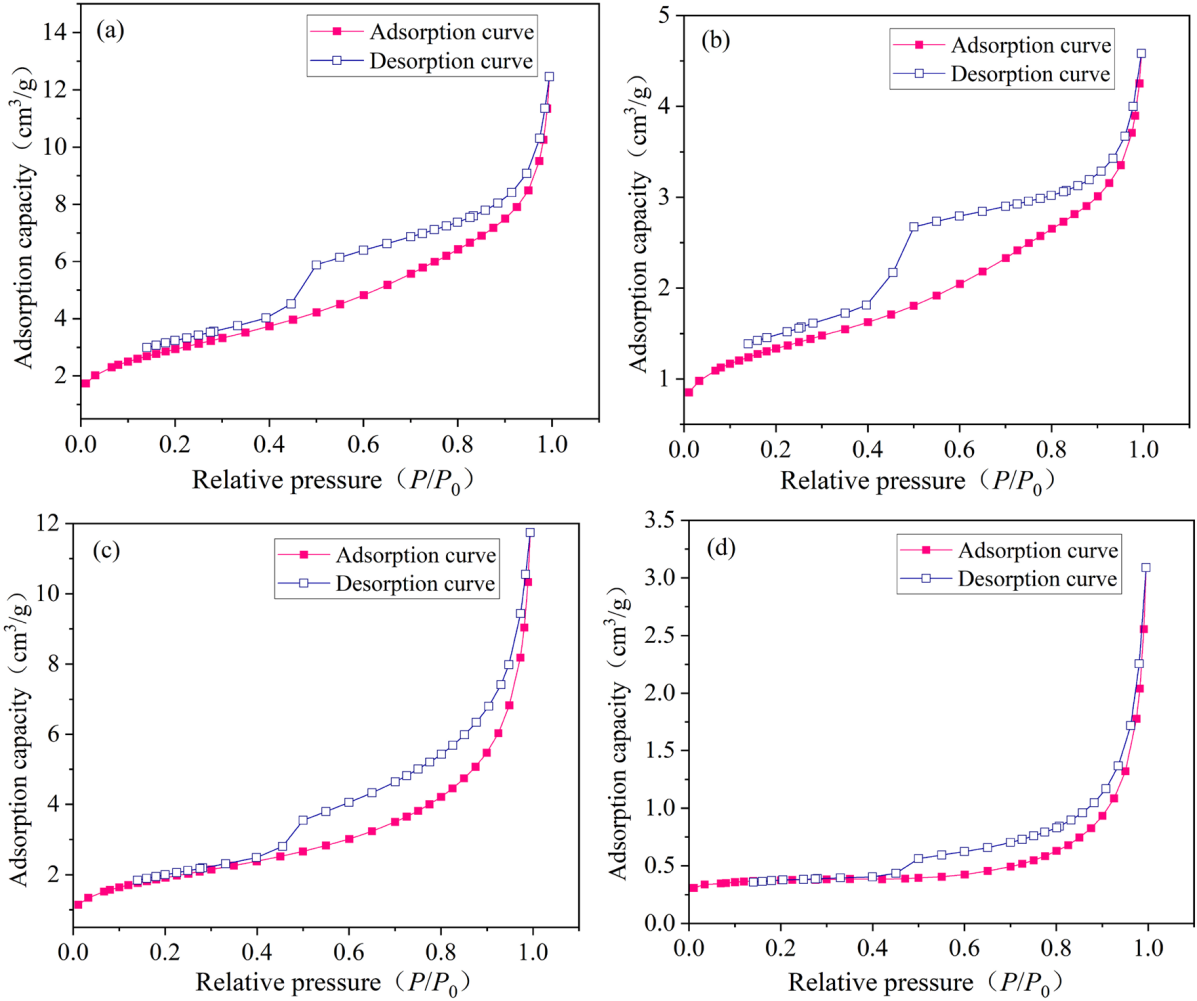
LP-N_2_ adsorption/desorption curves of vitrain and durain (**a**) YZG-JM, (**b**) YZG-AM, (**c**) HL-JM, (**d**) HL-AM.

The analysis of pore development characteristics of LP-N_2_ adsorption still adopts the IUPAC standard^48^. According to the LP-N_2_ adsorption/desorption isotherms of coal samples in Fig. 2, the adsorption isotherms of YZG and HL rose slowly in the low pressure area (*P*/*P*_0_ < 0.10), and the curve was slightly convex upward, which corresponded to the single molecular layer adsorption of N_2_ in the pore. In the medium pressure area (0.10 < *P*/*P*_0_ < 0.80), the adsorption capacity increased slowly with the increase of relative pressure, corresponding to the multi-molecular layer adsorption. Subsequently, the isothermal adsorption curve show an inflection point, and the adsorption capacity increased sharply in the higher pressure area (0.80 < *P*/*P*_0_ < 1), and the adsorption curve still showed an upward trend until *P*/*P*_0_ = 1, corresponding to the capillary condensation.

**Figure 3:**
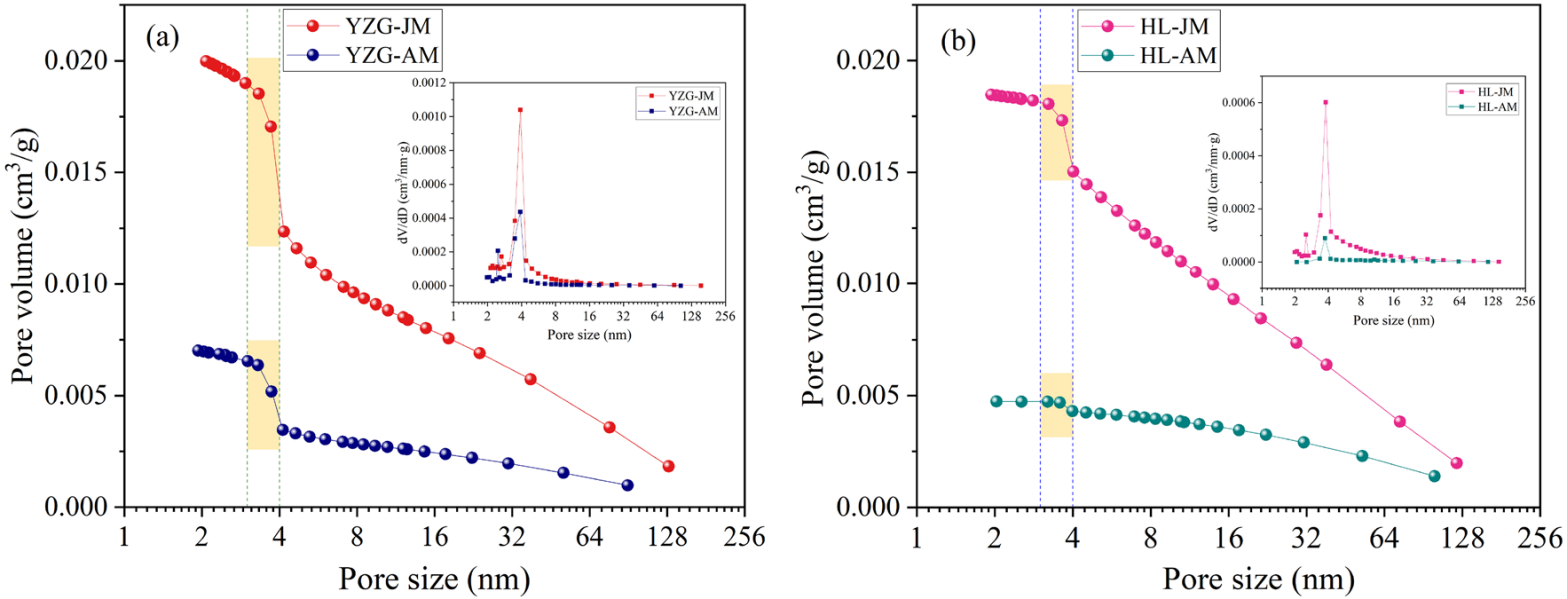
PV distribution of vitrain and durain with N_2_ adsorption (**a**) YZG, (**b**) HL.

**Figure 4:**
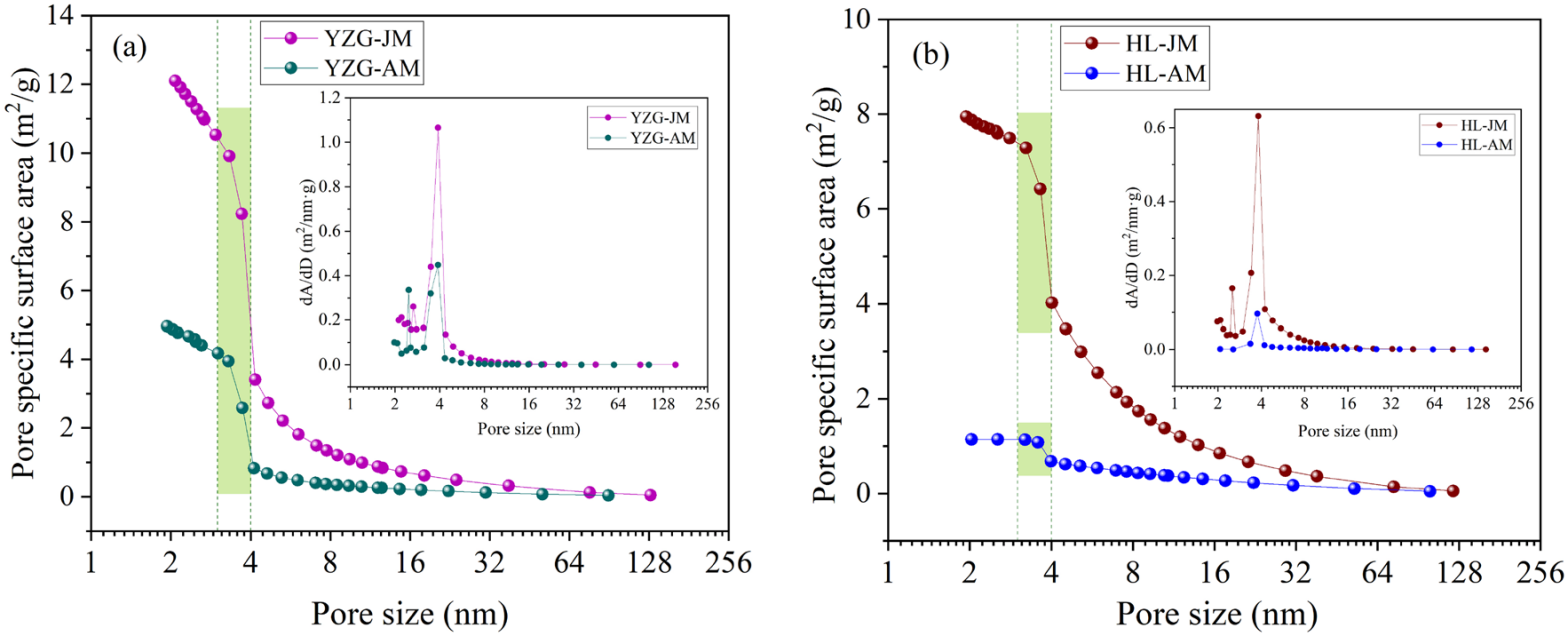
Pore SSA distribution of vitrain and durain with N_2_ adsorption (**a**) YZG, (**b**) HL.

The relationship between pore size and pore volume (PV) was shown in Fig. 3, the pore size is 1.8–145 nm, the PV curve of vitrain is higher than that of durain, and the PV increment of vitrain at each stage is greater than that of durain. The same pattern was observed between the pore SSA and the pore size (Fig. 4). The PV and pore SSA showed three stages of change. That is, increases slowly when the pore size is 4–145 nm. The PV and pore SSA increase significantly when the pore size is 3–4 nm. At pore size 1.8–3 nm, the slope of the curve slows down again. Comparing vitrain and durain in the same coal sample, it was found that the curve slope of vitrain was greater than that of durain, and the growth rate of vitrain was greater under the same pore size range. The PV and pore SSA growth curves 4–145 nm were analyzed, it was found that the PV curve could be approximated as a primary function, while the SSA curve was an exponential function.

Traditional BET and BJH models have certain deviation in the analysis of micropores materials. In addition to the BET and BJH models, the analysis of pore development characteristics was compared with the DFT model. It can be seen from Table 4, the pore SSA of DFT model was smaller than that of BET model, and the pore SSA of vitrain was obviously larger than that of durain in the same coal sample. The PV of the DFT model was smaller than that of the BJH model, and the PV of vitrain in the same coal sample was obviously larger than that of durain. The mean pore size of vitrain calculated by BET and BJH models was smaller than that of durain. Therefore, the analysis results of BET, BJH and DFT models were consistent for the pore characteristic parameters of vitrain and durain.

**Table 4:** Pore characteristics of vitrain and durain with N_2_ adsorption.

Coal sample	BET		BJH			DFT	
	SSA (m^2^/g)	Mean pore size (nm)	SSA (m^2^/g)	Mean pore size (nm)	PV (cm^3^/g)	SSA (m^2^/g)	PV (cm^3^/g)
YZG-JM	10.606	5.531	12.295	6.513	0.0201	2.414	0.00771
YZG-AM	4.756	6.615	4.952	5.668	0.0071	0.978	0.00329
HL-JM	6.894	9.271	8.008	9.240	0.0185	0.989	0.00747
HL-AM	1.269	12.458	1.144	16.558	0.0047	0.290	0.00434

### ***Pore fractal characteristics of vitrain and durain with LP-N***_***2***_*** adsorption***

According to the LP-N_2_ adsorption/desorption curve, there is an obvious hysteresis loop in the adsorption/desorption curve when the relative pressure *P*/*P*_0_ = 0.5, and the adsorption/desorption curves tend to coincide when *P*/*P*_0_ < 0.5. According to the principle of N_2_ adsorption in coal pores, when the relative pressure is low (*P*/*P*_0_ < 0.5), N_2_ molecules are mainly affected by the Vander Waals force on the surface of coal as monolayer adsorption. The fractal dimension at this stage represents the roughness of the pore surface, and the fractal dimension *D*_1_ is recorded. With the gradual increase of relative pressure (*P*/*P*_0_ > 0.5), N_2_ molecules adsorbed from single molecular layer to multi-molecular layer, from micropores to mesoporous and macropores, and the adsorption force gradually evolved from Vander Waals force to capillary condensation. The fractal dimension at this stage represents the complexity inside the pores, and as the fractal dimension *D*_2_. Therefore, the N_2_ adsorption/desorption curve is segmented, and the general pore fractal dimension is 2–3. The more tends to 2, the smoother and more simple it is, and the more tends to 3, the coarser and more complex it is.

**Figure 5:**
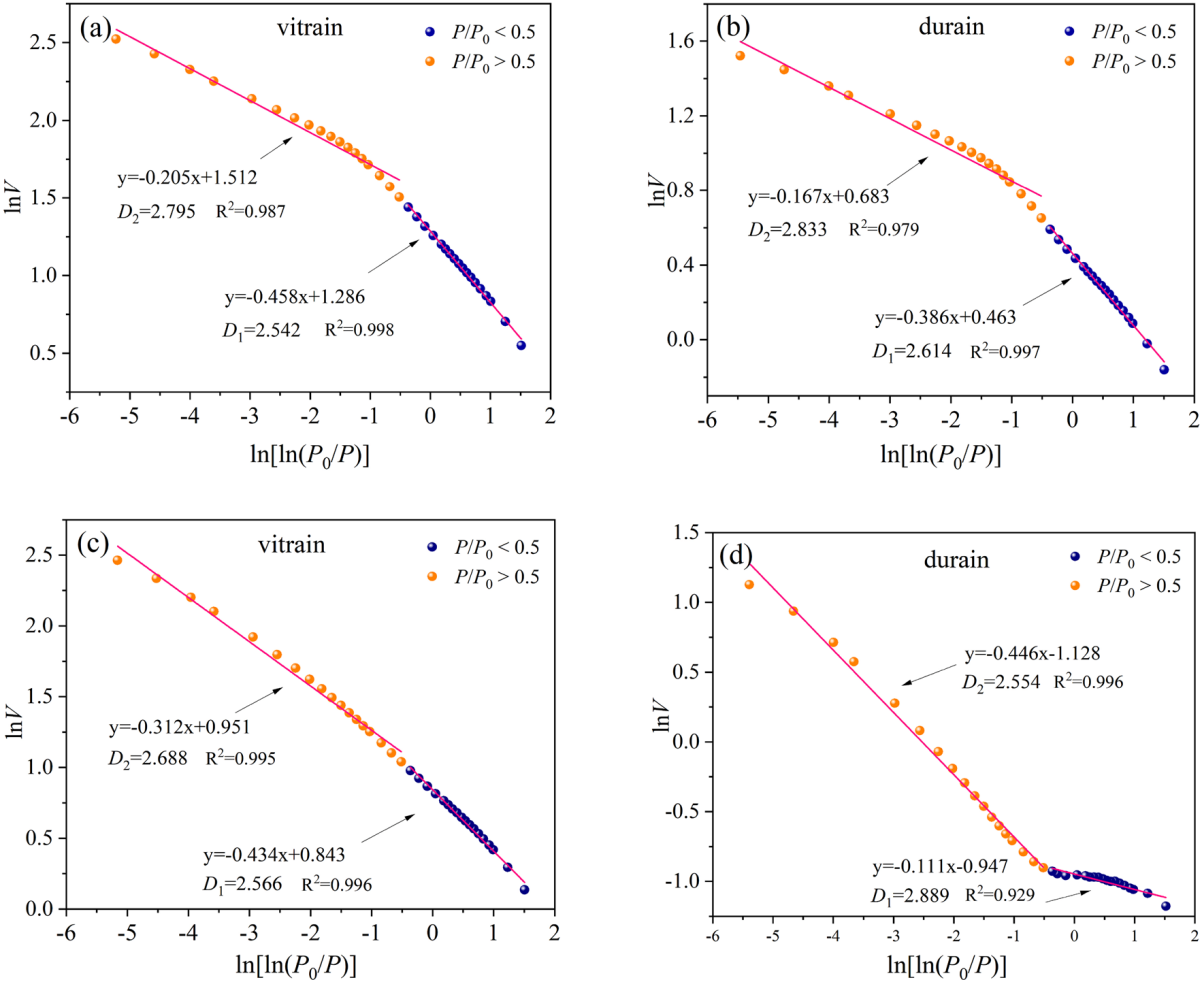
Pores fractal of vitrain and durain based on LP-N_2_ adsorption (**a**) YZG-JM, (**b**) YZG-AM, (**c**) HL-JM, (**d**) HL-AM.

The fractal dimension for LP-N_2_ adsorption/desorption were shown in Fig. 5. The fractal dimension *D*_1_ of YZG-JM and YZG-AM are 2.614 and 2.542, respectively, and the fractal dimension *D*_1_ of HL-JM and HL-AM are 2.889 and 2.566, respectively. The fractal dimension *D*_1_ of vitrain is greater than that of durain, indicating that the pore surface of vitrain is coarser than that of durain at the single molecular layer adsorption. When *P*/*P*_0_ > 0.5 is used for multilayer adsorption and capillary condensation, the fractal dimensions *D*_2_ of YZG-JM and YZG-AM are 2.833 and 2.795, and the fractal dimensions *D*_2_ of HL-JM and HL-AM are 2.554 and 2.688, respectively. The fractal dimension *D*_2_ of YZG-JM is larger than YZG-AM, while the fractal dimension *D*_2_ of HL-JM is smaller than HL-AM. On average, the fractal dimension *D*_2_ of durain is larger than vitrain, indicating that the pore structure of durain is more complex than vitrain.

### Macropore development characteristics of vitrain and durain

**Figure 6:**
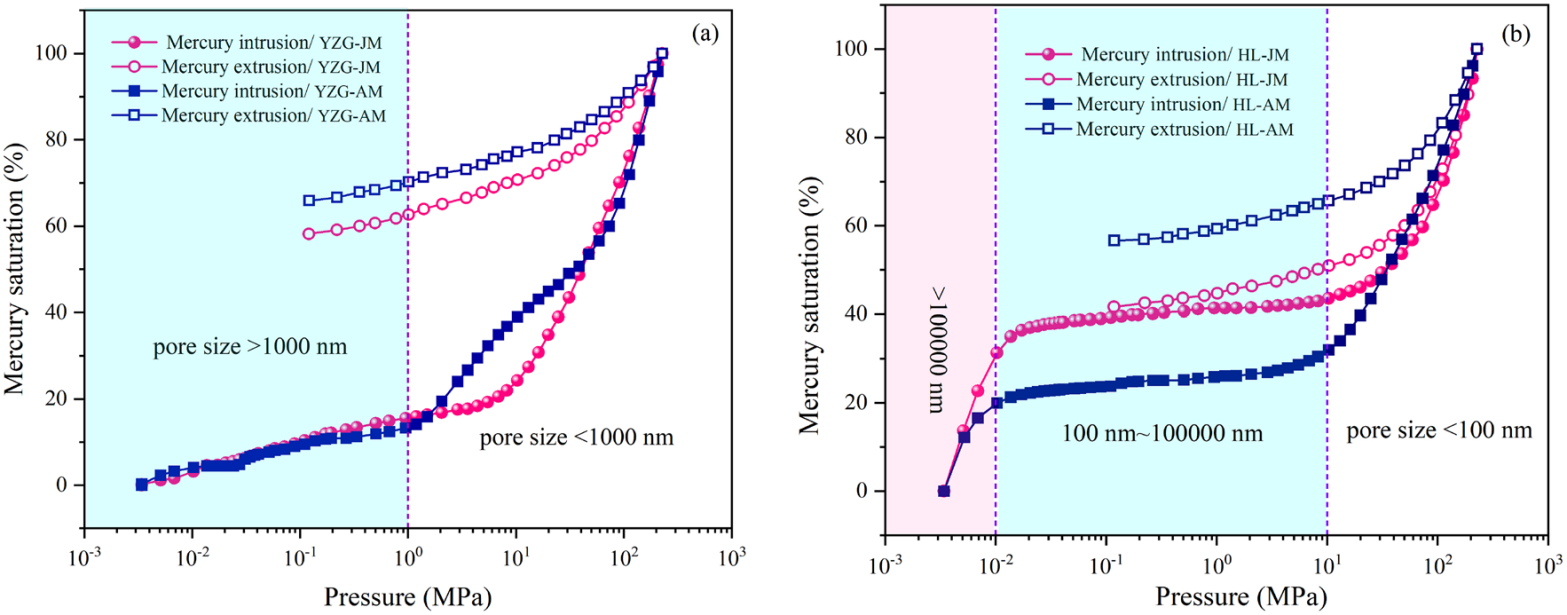
MIP curves of vitrain and durain (**a**) YZG, (**b**) HL.

As shown in Fig. 6, The pressure-mercury saturation curves of coal samples from YZG and HL showed obvious difference. The mercury intrusion curve of YZG can be divided into two section, pore size < 1000 nm is more developed, but there is little difference between vitrain and durain overall. HL coal sample can be divided into three section, the fracture (> 100,000 nm) and pore (< 100 nm) developed in vitrain and durain. Comparatively speaking, the fracture of vitrain is more developed.

**Figure 7:**
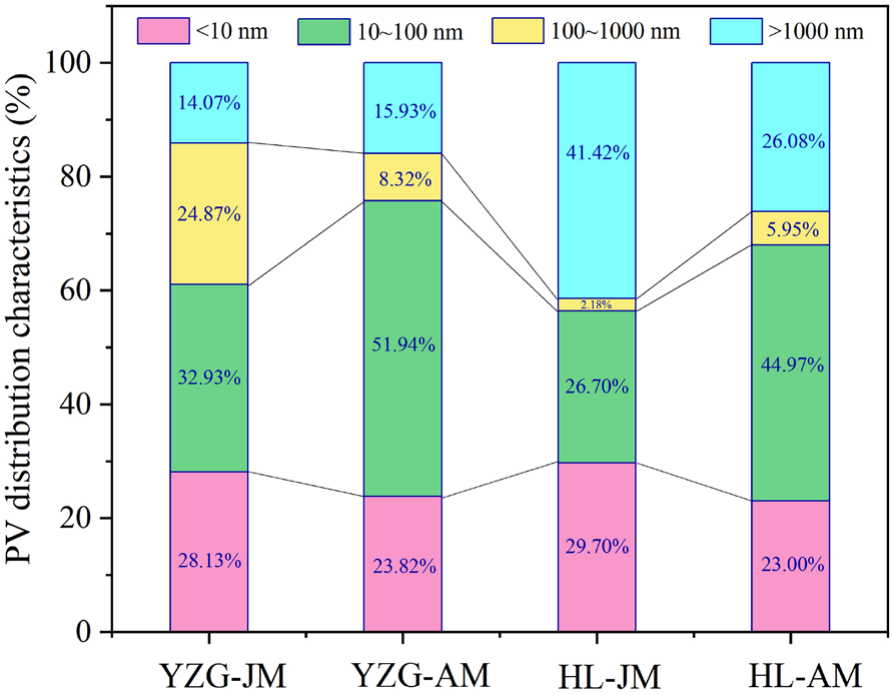
PV distribution of vitrain and durain with MIP.

**Figure 8:**
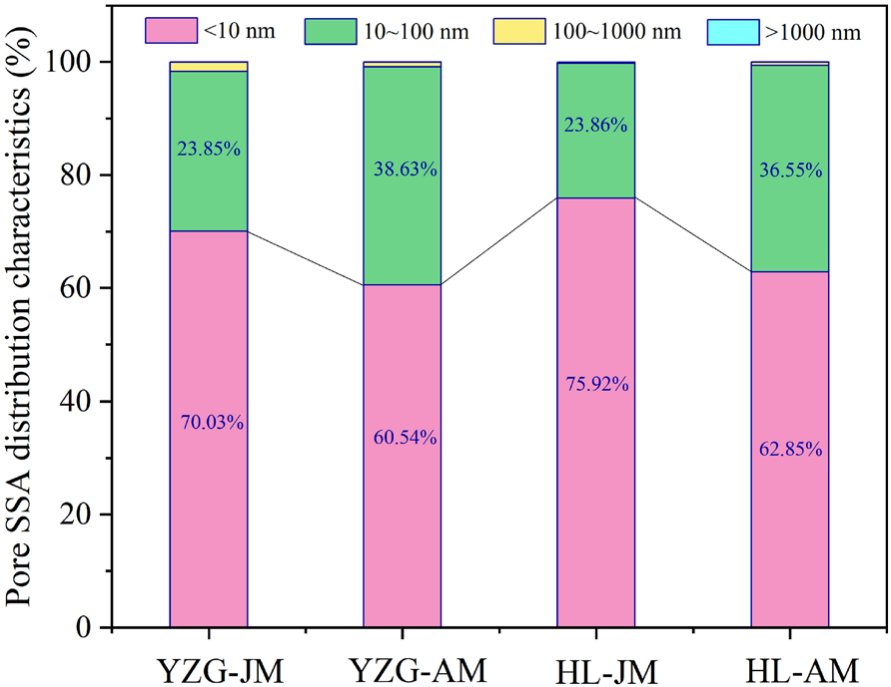
Pore SSA distribution of vitrain and durain with MIP.

The MIP range is limited (0.005–1000 μm), and the MIP could not characterize the pores with pore size < 2 nm, so the MIP adopted the pore size classification standard of B.B.Xoдoт. According to the results of MIP, the PV proportion of macropore and mesopore (> 100 nm) in YZG-JM are 38.94% and that of YZG-AM are 24.25%. HL-JM and HL-AM are 43.60% and 32.03%, respectively (Fig. 7). It is particularly noted that the macropore (micro-fractures) of HL-JM account for 41.42%, mainly because HL-JM is brittle and the fractures are very developed. The PV proportion of transition pore in durain is 51.94% (YZG-AM) and 44.97% (HL-AM), which is larger than that of vitrain 32.93% (YZG-JM) and 26.70% (HL-JM). The micropore development of vitrain is higher than that of durain in the same coal sample. In terms of SSA, micropores are still the first contributor of SSA, accounting for more than 60%. It is found that the micropore SSA of vitrain is 70.03% (YZG-JM) and 75.92% (HL-JM) than that of durain is 60.54% (YZG-AM) and 62.85% (HL-AM), and the transition pore SSA of durain is greater than that of vitrain (Fig. 8). The SSA of mesopore and macropore are so small that they are almost negligible.

Mercury intrusion/extrusion curve of YZG coal sample has a large opening and obvious hysteresis, which indicates that YZG is dominated by open pores and has good connectivity. The mercury extrusion efficiency of four coal samples ranges from 34.71 to 58.38% (Table 5), which does not exceed 60%, and is generally poor. The mercury extrusion curves are obviously different between HL-JM and HL-AM. The mercury extrusion efficiency of HL-JM is high (58.38%), reflecting that except for macropores (fracture), the other pores of HL-JM are mainly semi-open pores, and the connectivity is poor. HL-AM the mercury extrusion efficiency is 43.39%, and the mercury extrusion curve hysteresis is obvious at the middle and low pressure stage, indicating poor connectivity in the micropore-transition pore and good connectivity in the mesopore-macropore. In comparison, the mercury extrusion efficiency of HL-JM is maximum, mainly due to its fractures development, which is consistent with the research results of bright coal with rich fractures and good connectivity proposed by Qu et al^49^. According to the pore characteristics with MIP (Table 5), the threshold pressure and tortuosity of vitrain are greater than that of durain, but the mean pore size of vitrain is smaller than that of durain, and the pore structure of vitrain is more complex.

**Table 5:** Pore characteristics of vitrain and durain with MIP.

Coal sample	Mean pore size (nm)	Porosity (%)	Threshold pressure (KPa)	Tortuosity (%)	Mercury extrusion efficiency (%)
YZG-JM	19.10	11.21	29.85	8.443	34.17
YZG-AM	19.55	8.91	9.17	3.289	41.82
HL-JM	18.60	4.54	4.27	2.135	58.38
HL-AM	20.59	6.25	3.59	1.985	43.39

### Pore fractal characteristics of vitrain and durain with MIP

**Figure 9:**
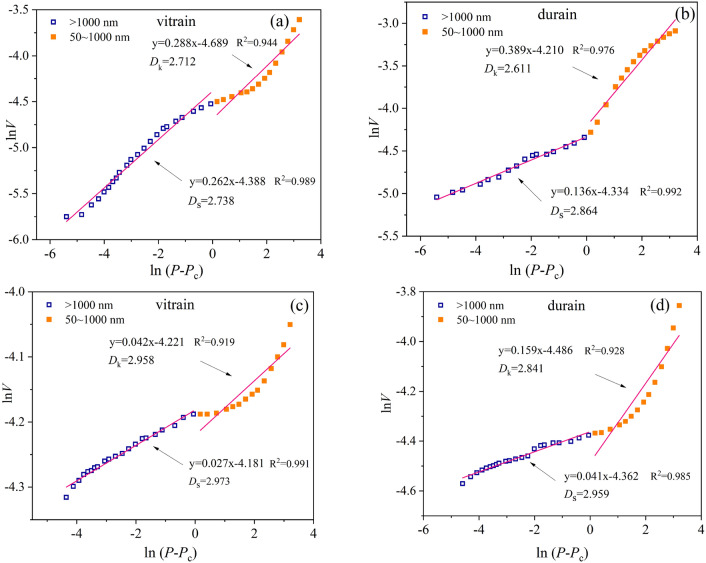
Pores fractal of vitrain and durain based on MIP (**a**) YZG-JM, (**b**) YZG-AM, (**c**) HL-JM, (**d**) HL-AM.

Macropores are mainly used as diffusion and permeability channels in the process of desorption and migration of CBM. However, it is insufficient to reflect the complexity of pore structure only by direct results of MIP. Fractal theory can make up for this defect, that is, to quantitatively express the complex problem of pore structure characteristics. LP-N_2_ absorption/desorption is mainly targeted at < 50 nm pores, so it is feasible to calculate the fractal characteristics of > 50 nm pores by MIP data. The pores with pore size > 50 nm were divided into macropore with pore size 50–1000 nm and fractures with pore size > 1000 nm. The diffusion capacity and permeability capacity of the pores were characterized, and the fractal dimensions are *D*_k_ and *D*_s_, respectively. According to the calculation results of fractal dimension (Fig. 9), the fractal dimension *D*_k_ of YZG-JM and YZG-AM are 2.712 and 2.611, respectively, and the fractal dimension *D*_k_ of HL-JM and HL-AM are 2.958 and 2.841, respectively, when the pore size is 50–1000 nm. The fractal dimension *D*_k_ of vitrain is larger than that of durain, indicating that the diffusion ability of macropore of vitrain is weak. In the range > 1000 nm, the fractal dimension *D*_s_ of YZG-JM and YZG-AM are 2.738 and 2.864, respectively, and the fractal dimension *D*_s_ of HL-JM and HL-AM are 2.973 and 2.959, respectively. The mean fractal dimension *D*_s_ of vitrain is smaller than that of durain, it indicates that vitrain has stronger seepage capacity, which is closely related to the fractures development of vitrain.

According to the above analysis results, it can be believed that there are obvious differences in pore development and pore structure of macro petrographic composition represented by vitrain and durain in low-rank coal reservoirs. Under the influence of coal-forming environment, vitrain is formed in water-covered sedimentary environment, and micropores develop. Later, due to the action of tectonic stress, vitrain brittleness leads to its fracture development. The durain is formed in dry oxidizing environment, transition pore development, the type of pore is mainly plant cell pore. Coalbed methane is stored in pores-fracture. Since the pore volume and specific surface area of vitrain are larger than that of durain and the micropore surface is rough, it is more favorable for methane adsorption and enrichment. The mean pore size of durain is larger than that of vitrain and the transition pore are develop and well connected, and the methane diffusion ability in macropores is strong, which is more conducive to the desorption and migration of methane.

## Conclusion

1. The basic material composition of vitrain and durain is different in the same coal sample. Vitrain has a higher volatile yield than durain, but the ash yield and fixed carbon content of durain are higher than that of vitrain Vitrain is rich in vitrinite, durain is rich in inertinite, and the content of liptinite is generally low in low-rank coal.
2. The micropores developed in vitrain and transitional pores developed in durain. The SSA and PV of vitrain are larger than that of durain, in which the micropore SSA of vitrain being 35% higher than that of durain. Transition pores and micro-fractures are the main contributors to the PV, accounting for more than 50% of the total PV. The LP-N_2_ absorption results showed that the PV curve could be approximated as a primary function, while the SSA curve was an exponential function when the pore size is 4–145 nm.
3. The mercury extrusion efficiency of coal samples is between 34.71 and 58.38%, which does not exceed 60%. In comparison, the mercury extrusion efficiency of HL-JM is maximum at 41.42%, which is mainly due to the development of micro-fractures. The threshold pressure and tortuosity of vitrain are greater than that of durain, but the mean pore size of vitrain is smaller than that of durain at different pore sizes.
4. Based on the calculation of the fractal dimension of pores with LP-N_2_ adsorption, it was found that at *P*/*P*_0_ < 0.5, the fractal dimension *D*_1_ of vitrain is larger than that of durain, indicating that the pore surface of vitrain is coarser than that of durain. when *P*/*P*_0_ > 0.5, the fractal dimension *D*_2_ of durain is larger than that of vitrain indicating that the pore structure of durain is more complex than that of vitrain. According to the results of MIP, in the range of pore size 50–1000 nm, the fractal dimension *D*_k_ of vitrain is larger than that of durain, it indicates that the diffusion ability of vitrain is weak. At the pore size > 1000 nm, the mean fractal dimension *D*_s_ of vitrain is smaller than that of durain, indicating that vitrain has developed fractures and strong seepage capacity.

## Data Availability

All data generated or analysed during this study are included in this published article.
